# Tick salivary gland components dampen Kasokero virus infection and shedding in its vertebrate reservoir, the Egyptian rousette bat (*Rousettus aegyptiacus*)

**DOI:** 10.1186/s13071-023-05853-7

**Published:** 2023-07-24

**Authors:** Amy J. Schuh, Brian R. Amman, Jonathan C. Guito, James C. Graziano, Tara K. Sealy, Jonathan S. Towner

**Affiliations:** 1 Viral Special Pathogens Branch, Division of High-Consequence Pathogens and Pathology, National Center for Emerging and Zoonotic Infectious Diseases, United States Centers for Disease Control and Prevention, Atlanta, GA USA; 2 United States Public Health Service Commissioned Corps, Rockville, MD USA

**Keywords:** Viruses, Chiroptera, Ticks, Saliva, Infection, Virus, Host interactions, Infectious disease reservoir, Zoonoses, Insect vectors, Vector-borne diseases

## Abstract

**Background:**

The human-pathogenic Kasokero virus (KASV) circulates in an enzootic transmission cycle between Egyptian rousette bats (ERBs; *Rousettus aegyptiacus*) and their argasid tick ectoparasites, *Ornithodoros (Reticulinasus) faini*. Although tick salivary gland components have been shown to potentiate virus infection in vertebrate non-reservoirs (i.e. incidental hosts or small animal models of disease), there is a lack of information on the effect of tick salivary gland components on viral infection and shedding in vertebrate reservoirs.

**Methods:**

To determine the impact of tick salivary gland components on KASV infection and shedding in ERBs, KASV loads were quantified in blood, oral swab, rectal swab, and urine specimens collected daily through 18 days post inoculation from groups of ERBs intradermally inoculated with KASV or KASV + *O. (R.) faini* tick salivary gland extract (SGE).

**Results:**

Bats inoculated with KASV + tick SGE had significantly lower peak and cumulative KASV viremias and rectal shedding loads compared to bats inoculated with KASV only.

**Conclusions:**

We report for the first time to our knowledge that tick salivary gland components dampen arbovirus infection and shedding in a vertebrate reservoir. This study advances our understanding of biological factors underlying arbovirus maintenance in nature.

**Graphical abstract:**

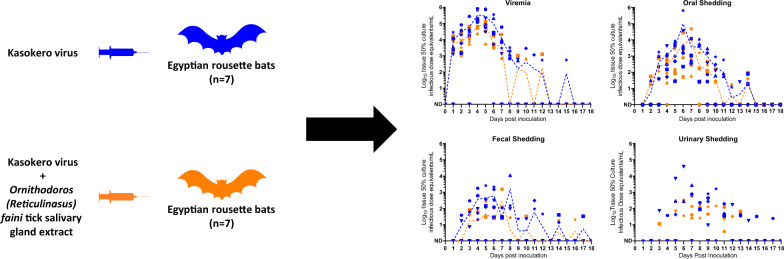

**Supplementary Information:**

The online version contains supplementary material available at 10.1186/s13071-023-05853-7.

## Background

Kasokero virus (KASV; family *Nairoviridae*, genus *Orthonairovirus*) was first described by scientists at the Uganda Virus Research Institute (UVRI) following the isolation of infectious virus (2.7% [2/74] bats) and detection of virus-specific antibodies (67.6% [50/74] bats) in serum samples collected from Egyptian rousette bats (ERBs; *Rousettus aegyptiacus*) captured at Kasokero Cave, Uganda, in 1977 [1]. During initial virus characterization efforts at UVRI, three laboratory staff and one support staff acquired KASV infection with clinical manifestations ranging from mild febrile illness to long-lasting systemic disease. Infectious KASV was later isolated from engorged and unengorged *Ornithodoros (Reticulinasus) faini* ticks collected from the rock crevices of ERB roosts at Lanner Gorge Cave, South Africa, in 1994–1995 and Python Cave, Uganda in 2017 [2]. Consistent with the expectations of a vertebrate reservoir of a virus, our group recently demonstrated that ERBs intradermally inoculated with a KASV dose within the range of viral loads detected in unengorged *O. (R.) faini* ticks became viremic and exhibited significant oral, fecal, and urinary shedding and high viral loads in the skin at the inoculation site, liver, inguinal lymph nodes, and spleen in the absence of overt clinical disease, followed by seroconversion [3].

Previous work has shown that tick salivary gland components, likely proteins or peptides [4], enhance virus infection in vertebrate non-reservoirs (i.e. incidental hosts and animal models of human disease). Labuda et al. (1993) demonstrated that 67% (6/9) of uninfected *Rhipicephalus appendiculatus* nymph-infested guinea pigs (animal model of human disease) inoculated with tick-borne encephalitis virus (TBEV; family *Flaviviridae*; genus *Flavivirus*) + tick salivary gland extract (SGE; derived from *Ixodes ricinus*, *Dermacentor reticulatus*, and *R. appendiculatus* ticks) developed viremia compared to 30% (3/10) of *R. appendiculatus* nymph-infested guinea pigs inoculated with TBEV alone [5]. In line with this observation, a higher percentage of recipient ticks acquired TBEV infection after feeding on the TBEV + tick SGE-inoculated guinea pigs (mean range: 28.6–51.7%) compared to recipient ticks that had fed on the guinea pigs inoculated with TBEV alone (9.8%). Hermance et al. (2015) demonstrated that 100% of BALB/c mice (small animal model of human disease) intradermally inoculated with the tick-borne encephalitic Powassan virus (POWV; family *Flaviviridae*, genus *Flavivirus*) survived and exhibited no clinical signs of disease, whereas 100% of mice intradermally inoculated with POWV + *Ixodes scapularis* tick SGE succumbed to infection [6]. Furthermore, the mice that also received tick SGE exhibited higher POWV loads in the blood, lymph nodes proximal to the inoculation site, and brain.

Although tick salivary gland components have been shown to potentiate virus infection in vertebrate non-reservoirs [5, 6], there is a lack of information on the effect of tick salivary gland components on viral infection and shedding dynamics in virus-natural vertebrate reservoir host systems. Herein, we determine the impact of tick salivary gland components on KASV infection and shedding dynamics in ERBs by inoculating groups of bats with KASV or KASV + *O. (R.) faini* tick SGE and then measuring KASV in blood, oral swab, rectal swab, and urine specimens collected through 18 days post inoculation (DPI).

## Methods

Salivary glands were dissected from live *O. (R.) faini* ticks collected with forceps from rock crevices of a large ERB colony (~ 40,000 individuals) at Python Cave, Queen Elizabeth National Park, Uganda, in December 2019. After ticks were rinsed with phosphate-buffered saline (PBS) and blotted dry with a lint-free cloth, they were embedded ventral side down in melted paraffin wax on a glass slide. Using a dissecting microscope, the dorsum was removed from each tick using a Feather Micro Scalpel 15° (VWR International, Wayne, PA, USA), and salivary glands were excised using micro-dissecting forceps. Salivary gland pairs were rinsed in a pool of PBS on a glass slide and then viewed under the microscope to ensure integrity. Pools of five salivary gland pairs were transferred to cryovials containing 0.5 ml PBS and then placed into liquid nitrogen. After the frozen tick salivary gland pools received 5 megarads of gamma irradiation while on dry ice, they were thawed on wet ice, transferred to grinding vials (OPS Diagnostics, Lebanon, NJ), homogenized using the GenoGrinder 2000 (OPS Diagnostics), pooled, and transferred to fresh cryovials. The protein concentration of the pooled tick SGE was measured using the Qubit Protein Assay Kit (Thermo Fisher Scientific, Waltham, MA, USA).

To mimic tick feeding, two groups of 5–7-month-old, captive-bred juvenile ERBs (each group included 4 males and 3 females) from the ERB breeding colony [9] were intradermally inoculated in the subcaudal abdominal region under isoflurane anesthesia with either: (1) 4.0 log_10_ tissue culture infectious dose 50% (log_10_TCID_50_) of the UGA-Tick-20170128 strain of KASV (Vero E6 + 2 passages; sequence identical to GenBank Accession Numbers MT309090, MT309094, and MT309097 [Vero E6 + 1]) prepared in 0.1 ml of sterile PBS (results previously reported in Schuh et al., 2022 [3]) or (2) 4.0 log_10_TCID_50_ of the UGA-Tick-20170128 strain of KASV + 100 µg gamma-irradiated, *O. (R.) faini* tick SGE (protein concentration equal to the concentration of extract made from one pair of salivary glands) prepared in 0.1 ml sterile PBS. Weights were taken from all bats on a weekly basis, and rectal temperatures, blood, oral swabs, rectal swabs, and urine (opportunistically) were collected daily through 18 DPI using previously described procedures [9, 10]. Bats were euthanized by an overdose of isoflurane followed by cardiac exsanguination at 18 and 20 DPI. Using an established assay [3], KASV loads in blood, oral swab, rectal swab, and urine specimens were monitored by qRT-PCR (assay standardized to KASV log_10_TCID_50_ equivalents (eq)/ml]). Anti-KASV IgG responses were measured using a previously described indirect enzyme linked immunosorbent assay [3].

The duration of viremia/viral shedding and peak viremia/shedding loads was determined for each sample type according to individual bat. Cumulative viremia and viral shedding are measures of infectiousness [11] and were calculated for each bat by summing KASV loads detected in the blood and oral and rectal swabs through 18 DPI. Prism 9.0.0 software (GraphPad, La Jolla, CA, USA) was used to test data normality and homoscedasticity assumptions and perform statistical hypothesis testing.

## Results and discussion

**Fig. 1:**
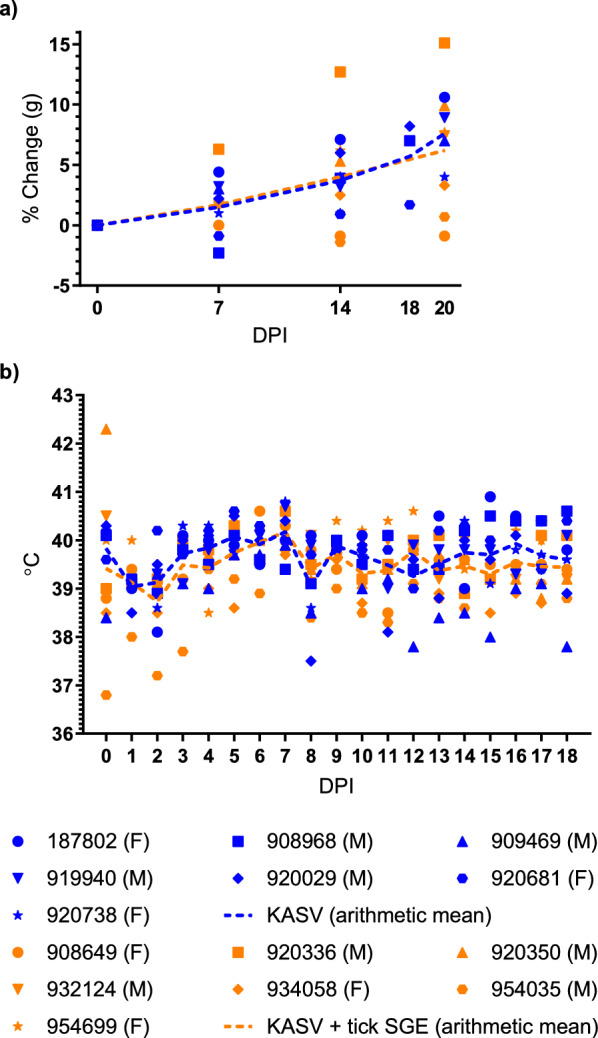
Weights and body temperatures. **a** Percent weight change from baseline at 0 days post inoculation (DPI) and **b** temperatures of Kasokero virus (KASV)- and KASV + tick salivary gland extract (SGE)-inoculated bats. Bat identification numbers correspond to numeric codes generated by scanning implanted passive integrated transponder tags

Although a previous serial sacrifice study showed that KASV-inoculated ERBs do experience KASV-induced acute, self-limiting hepatitis [3, 12], none of the KASV-inoculated or KASV + tick SGE-inoculated bats in this study exhibited overt signs of clinical disease at any time point. There were no statistically significant differences in mean weight change from baseline at 7 (multiple Mann-Whitney *U* tests, *U* = 14, Holm-Šídák-adjusted *P* = 0.239974) and 14 DPI (Mann-Whitney *U*, *U* = 14, Holm-Šídák-adjusted *P* = 0.239974) between the two bat groups (Fig. 1a) nor statistically significant differences in temperature at any time point from 0 to 18 DPI between the two bat groups (Fig. 1b; multiple unpaired t-tests, *t* ratio range 0.07994–3.348, *df* = 12, Holm-Šídák-adjusted *P* range 0.104676–0.99549).

**Fig. 2:**
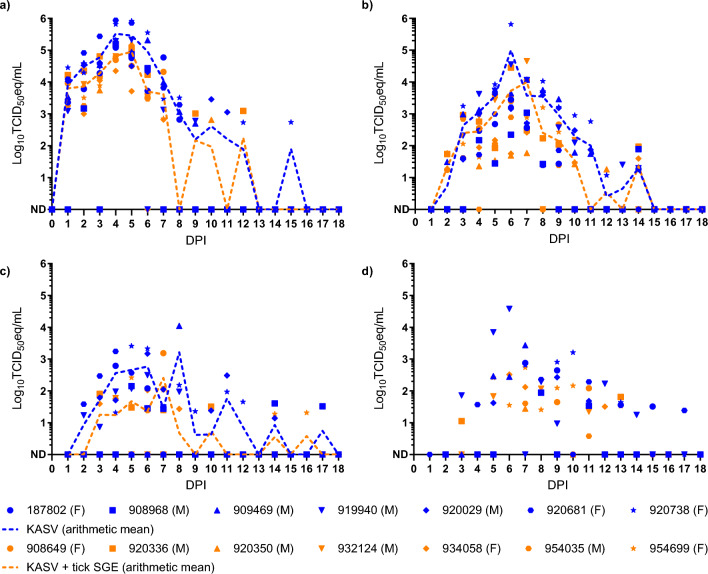
Kasokero virus (KASV) loads detected in bat specimens. KASV loads (qRT-PCR-derived log_10_ 50% tissue culture infectious dose equivalents per ml [log_10_TCID_50_eq/ml]) in **a** blood, **b** oral swabs, **c** rectal swabs, and **d** urine collected daily from KASV- and KASV + tick salivary gland extract (SGE)-inoculated bats through 18 days post inoculation (DPI). ND: Not detected. Bat identification numbers correspond to numeric codes generated by scanning implanted passive integrated transponder tags

KASV viremia (Fig. 2a), and oral (Fig. 2b), fecal (Fig. 2c), and urinary (Fig. 2d) shedding was detected in all bats from both groups at ≥ 1 time point during the study. KASV + tick SGE-inoculated bats had significantly lower peak viremia loads (Mann-Whitney U test, *U* = 7, *P* = 0.0262), cumulative viremia loads (unpaired t-test, *t* = 2.845, *df* = 12, *P* = 0.0148), peak rectal shedding loads (unpaired t-test, *t* = 2.926, *df* = 12, *P* = 0.0127), and cumulative rectal shedding loads (unpaired t-test, *t* = 3.299, *df* = 12, *P* = 0.0064) compared to KASV-inoculated bats (Table 1). Although the durations of viremia, oral shedding, and rectal shedding were shorter, and peak oral shedding loads and cumulative oral shedding loads were lower in KASV + tick SGE-inoculated bats than KASV-inoculated bats, these differences were not statistically significant (Table 1). The opportunistic nature of the urine collections prohibited statistical group comparisons of duration of shedding, peak shedding loads, and cumulative shedding loads; however, the KASV detection ratio in urine was not significantly lower in KASV + tick SGE-inoculated bats than KASV-inoculated bats (Table 1).

**Table 1:** Kasokero virus (KASV) infection and shedding dynamics in KASV-inoculated bats versus KASV + tick salivary gland extract (SGE)-inoculated bats

Parameter	KASV-inoculated bats, arithmetic mean (SD)^a^	KASV + tick SGE-inoculated bats, arithmetic mean (SD)^a^	Statistical hypothesis testing
Duration of viremia (days)	7.7 (1.9)	6.9 (0.7)	Welch’s t-test, Welch-corrected *t* = 1.127, *df* = 7.572, *P* = 0.2941
Peak viremia loads (log_10_TCID_50_eq/ml)	5.4 (0.4)^a^	4.9 (0.3)^a^	Mann-Whitney *U* = 7, *P* = 0.0262*
Cumulative viremia loads (log_10_TCID_50_eq/ml)	5.8 (0.4)^a^	5.2 (0.3)^a^	Unpaired t-test, *t* = 2.845, *df* = 12, *P* = 0.0148*
Duration of oral shedding (days)	8.6 (1.9)	7.3 (1.7)	Unpaired t-test, *t* = 1.332, *df* = 12, *P* = 0.2077
Peak oral shedding loads (log_10_TCID_50_eq/ml)	4.0 (1.0)^a^	3.5 (1.1)^a^	Unpaired t-test, *t* = 0.7603, *df* = 12, *P* = 0.4617
Cumulative oral shedding loads (log_10_TCID_50_eq/ml)	4.2 (0.9)^a^	3.8 (0.9)^a^	Unpaired t-test, *t* = 0.9088, *df* = 12, *P* = 0.3813
Duration of rectal shedding (days)	4.9 (2.5)	2.6 (1.3)	Unpaired t-test, *t* = 2.571, *df* = 12, *P* = 0.0507
Peak rectal shedding loads (log_10_TCID_50_eq/ml)	3.0 (0.6)^a^	2.1 (0.5)^a^	Unpaired t-test, *t* = 2.926, *df* = 12, *P* = 0.0127*
Cumulative rectal shedding loads (log_10_TCID_50_eq/ml)	3.2 (0.6)^a^	2.3 (0.5)^a^	Unpaired t-test, *t* = 3.299, *df* = 12, *P* = 0.0064*
Detection ratio in urine (detections/collections)	0.48 (0.17)	0.44 (0.12)	Unpaired t-test, *t* = 0.4967, *df* = 12, *P* = 0.6284

^a^If KASV- and KASV + tick SGE-inoculated bat datasets were lognormally distributed, geometric means and geometric standard deviations were calculated, and statistical hypothesis testing was performed on log-transformed datasets; otherwise, arithmetic means and standard deviations were calculated, and no data transformations were performed prior to statistical hypothesis testing. SD: standard deviation, log_10_TCID_50_eq/ml: log_10_ 50% tissue culture infectious dose equivalents per ml; *t*: test statistic, *df*: degrees of freedom, and *P*: probability

All KASV-inoculated and KASV + tick SGE-inoculated bats seroconverted to KASV by 14 DPI (Additional file 1: Figure S1); small group sizes at 18 and 20 DPI precluded group statistical comparisons of anti-KASV IgG antibody responses.

In contrast to previous work showing that tick SGE potentiates arbovirus infections in non-reservoir vertebrate hosts [5, 6], we demonstrated that *O. (R.) faini* tick SGE dampens KASV viremia and rectal shedding in ERBs, natural vertebrate reservoir hosts for KASV. Although we are not aware of any studies examining the effect of tick SGE on arbovirus infection dynamics in natural vertebrate reservoir hosts, Park et al. (2021) demonstrated that domestic pigs (natural amplification hosts) inoculated with Japanese encephalitis virus (JEV; family *Flaviviridae*, genus *Flavivirus*) + *Culex quinquefasciatus* mosquito SGE exhibited milder febrile illnesses and shortened durations of nasal shedding compared to pigs inoculated with JEV alone [13]. Similar to Park et al. [13], we speculate that vertebrate reservoirs of arboviruses may respond differently to tick saliva than vertebrate non-reservoirs. From an evolutionary perspective, controlled arbovirus replication and low vertebrate reservoir mortality would help ensure long-term maintenance of the virus in nature.

Previous studies investigating the effect of tick salivary gland components on virus infection in small animal models of human disease used SGE prepared from colonized ticks [5, 6]. Due to the non-existence of an *O. (R.) faini* tick colony, we sterilized the frozen tick salivary gland pools by subjecting them to 5 megarads of gamma irradiation while on dry ice to ensure our study investigated the effect of protein and peptides in tick SGE on KASV infection in ERBs, not the synergistic effect of tick SGE and uncharacterized microbes present in the SGE on virus infection. Notably, previous studies have demonstrated that low temperatures (i.e. dry ice [~ − 80 °C]) protect the structural stability and functional activity of proteins in aqueous solutions subjected to high-dose gamma irradiation (5 megarads) [7, 8].

In conclusion, we report for the first time to our knowledge that tick salivary gland components dampen arbovirus infection and shedding in a vertebrate reservoir. This study advances the understanding of biological factors that influence arbovirus maintenance in nature.

## Supplementary Information

**Additional file 1: Fig. S1**. Anti-Kasokero virus (KASV) IgG responses. Whole blood for serology was collected at 0, 7, and 14 DPI and at the end of the study (18 or 20 DPI) from KASV- and KASV + tick salivary gland extract (SGE)-inoculated bats. Bat identification numbers correspond to numeric codes generated by scanning implanted passive integrated transponder tags.

## Data Availability

All data generated or analyzed during this study are included in this published article and its supplementary information file.

## Abbreviations

KASV: Kasokero virus
ERB: Egyptian rousette bat
*O*. (*R*.) *faini*: *Ornithodoros * (*Reticulinasus*) * faini*
TBEV: Tick-borne encephalitis virus
SGE: Salivary gland extract
POWV: Powassan virus
DPI: Days post inoculation
PBS: Phosphate-buffered saline
log_10_TCID_50_: Log_10_ tissue culture infectious dose 50%
Eq: Equivalents
*P*: Probability
*t*: *t*-test
*df*: Degrees of freedom
JEV: Japanese encephalitis virus

## References

[1] Kalunda M, Mukwaya LG, Mukuye A, Lule M, Sekyalo E, Wright J (1986). Kasokero virus: a new human pathogen from bats (*Rousettus aegyptiacus*) in Uganda. Am J Trop Med Hyg.

[2] Schuh AJ, Amman BR, Patel K, Sealy TK, Swanepoel R, Towner JS (2020). Human-pathogenic Kasokero virus in field-collected ticks. Emerg Infect Dis.

[3] Schuh AJ, Amman BR, Guito JC, Graziano JC, Sealy TK, Kirejczyk SGM (2022). Natural reservoir *Rousettus aegyptiacus* bat host model of orthonairovirus infection identifies potential zoonotic spillover mechanisms. Sci Rep.

[4] Jones LD, Hodgson E, Nuttall PA (1991). Characterization of tick salivary gland factor(s) that enhance Thogoto virus transmission. Hemorrhagic fever with renal syndrome, tick- and mosquito-borne viruses.

[5] Labuda M, Jones LD, Williams T, Nuttall PA (1993). Enhancement of tick-borne encephalitis virus transmission by tick salivary gland extracts. Med Vet Entomol.

[6] Hermance ME, Thangamani S (2015). Tick Saliva Enhances Powassan Virus Transmission to the Host, Influencing Its Dissemination and the Course of Disease. J Virol.

[7] Smeltzer CC, Lukinova NI, Towcimak ND, Yan X, Mann DM, Drohan WN (2015). Effect of gamma irradiation on the structural stability and functional activity of plasma-derived IgG. Biologicals.

[8] David SC, Lau J, Singleton EV, Babb R, Davies J, Hirst TR (2017). The effect of gamma-irradiation conditions on the immunogenicity of whole-inactivated Influenza A virus vaccine. Vaccine.

[9] Amman BR, Jones ME, Sealy TK, Uebelhoer LS, Schuh AJ, Bird BH (2015). Oral shedding of Marburg virus in experimentally infected Egyptian fruit bats (*Rousettus aegyptiacus*). J Wildl Dis.

[10] Schuh AJ, Amman BR, Jones ME, Sealy TK, Uebelhoer LS, Spengler JR (2017). Modelling filovirus maintenance in nature by experimental transmission of Marburg virus between Egyptian rousette bats. Nat Commun.

[11] Chase-Topping M, Gally D, Low C, Matthews L, Woolhouse M (2008). Super-shedding and the link between human infection and livestock carriage of *Escherichia coli* O157. Nat Rev Microbiol.

[12] Kirejczyk SGM, Schuh AJ, Zhang J, Amman BR, Guito JC, Sealy TK (2023). Pathogenesis of Kasokero virus in experimentally infected Egyptian rousette bats (*Rousettus aegyptiacus*). Vet Pathol.

[13] Park SL, Huang Y-JS, Lyons AC, Ayers VB, Hettenbach SM, McVey DS, et al. Mosquito saliva modulates Japanese encephalitis virus infection in domestic pigs. Front Virol. 2021;1. 10.3389/fviro.2021.724016.

[14] National Research Council. Guide for the Care and Use of Laboratory Animals: Eighth Edition. Washington, DC: The National Academies Press; 2011. 246 p.

