# Supplementary material for: Tick salivary gland components dampen Kasokero virus infection and shedding in its vertebrate reservoir, the Egyptian rousette bat (Rousettus aegyptiacus)
Source: Parasit Vectors. 2023 Jul 24;16:249. doi: 10.1186/s13071-023-05853-7 (PMC10367358; doi:10.1186/s13071-023-05853-7)
Supplement: Supplementary file 1 — Additional file 1: Fig. S1. Anti-Kasokero virus (KASV) IgG responses. Whole blood for serology was collected at 0, 7, and 14 DPI and at the end of the study (18 or 20 DPI) from KASV- and KASV + tick salivary gland extract (SGE)-inoculated bats. Bat identification numbers correspond to numeric codes generated by scanning implanted passive integrated transponder tags. [file 13071_2023_5853_MOESM1_ESM.pdf]

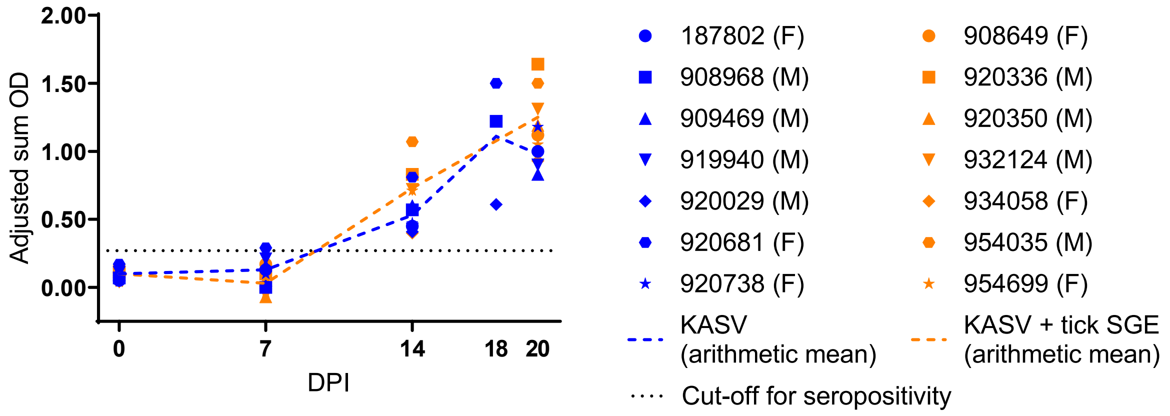

Fig. S1. Anti-Kasokero virus (KASV) IgG responses. Whole blood for serology was collected at 0, 7, and 14 DPI and at the end of the study (18 or 20 DPI) from KASV- and KASV + tick salivary gland extract (SGE)-inoculated bats. Bat identification numbers correspond to numeric codes unique to each implanted passive integrated transponder tag.
